# Clinical features and prognostic indicators in upper-tract urothelial carcinoma with bone metastasis

**DOI:** 10.3389/fsurg.2022.928294

**Published:** 2022-08-30

**Authors:** Mingping Zhou, Jianxin Zhang, Xiaowei Chen, Zhan Wang, Wei Liang

**Affiliations:** ^1^ Department of Orthopedics, Lishui City People's Hospital, The Sixth Affiliated Hospital of Wenzhou Medical University, The First Affiliated Hospital of Lishui University, Lishui, China; ^2^ Department of Orthopedics, Qingtian People’s Hospital, Qingtian, China; ^3^ Department of Orthopedics, People’s Hospital of Jing Ning She Autonomous County, Jingning, China; ^4^ Department of Orthopedic Surgery, The Second Affiliated Hospital, Zhejiang University School of Medicine, Hangzhou, China

**Keywords:** upper-tract urothelial carcinoma (UTUC), renal pelvis, ureter, bone metastasis, survival, risk factor

## Abstract

**Purpose:**

With the gradual increase in the incidence of upper-tract urothelial carcinoma (UTUC), its metastatic disease has attracted much attention. The prognosis of UTUC patients with bone metastasis is worse than that of UTUC patients with other metastases. Therefore, the current study is performed to analyze the clinicopathologic features and survival predictors among UTUC patients with bone metastasis.

**Patients and **m**ethods:**

We reviewed the Surveillance, Epidemiology, and End Results (SEER) database to select cases diagnosed with UTUC and bone metastasis at present from 2010 to 2016. Overall survival (OS) and cancer-specific survival (CSS) were first performed by applying univariate Cox regression analysis. Then we performed multivariate Cox analysis to determine independent predictors of survival. Forest plots were drawn by GraphPad 8.0.1 and used to visually display the results of multivariate analysis. Kaplan-Meier method was applied to intuitively show the prognosis difference of each independent risk factor.

**Results:**

We finally identified 380 UTUC patients with bone metastasis for survival analysis, of which 230 males (60.5%) and 150 females (39.5%). The mean and median age at diagnosis were 71 and 72 years, respectively. Simultaneous lung metastasis (33.4%) and liver metastasis (31.1%) were more common in UTUC patients with bone metastasis. The 1-year OS and CSS rates of this research population were 23.8% and 26.6%, respectively. Multivariate Cox proportional hazards modeling controlling for surgery, chemotherapy, brain metastasis, liver metastasis, lung metastasis, and marital status, revealed that surgery, chemotherapy, no liver metastasis, no lung metastasis, and married status predicted for better OS and CSS.

**Conclusion:**

Surgery and chemotherapy are optimal management of UTUC patients with bone metastasis. Active treatments on lung and liver metastases should be performed. The prognosis of patients with unmarried status or others should be further improved.

## Introduction

Upper-tract urothelial carcinoma (UTUC) is a relatively uncommon disease of the urinary system, accounting for 5%–10% of all urothelial carcinomas (1). UTUC includes carcinoma of the renal pelvis and ureter (2, 3). Metastasis was observed in about 7% of UTUC patients and 3-year overall survival (OS) rates for metastatic UTUC were less than 10% (4). Compared to other metastatic sites, bone metastasis had less favorable prognosis (5). With the gradual increase in the incidence of UTUC (1) and its invasiveness at diagnosis (6), more and more studies have been conducted to analyze its prognosis (1, 7, 8). Giving the rarity of UTUC with bone metastasis, clinicopathologic features, epidemiological factors, and survival data are absent.

Mainstream treatments of UTUC include surgical resection and chemotherapy (9). Although there have been many studies on the treatment of UTUC, there have been few studies on metastatic UTUC, especially UTUC with bone metastasis. Whether surgical management and chemotherapy can prolong the survival of UTUC with bone metastasis remains unknown. Radiotherapy is usually used as one of the palliative treatments for patients with advanced tumors. Whether radiotherapy is also suitable for UTUC with bone metastasis remains to be further explored.

To obtain insight into UTUC with bone metastasis, we used data from the Surveillance, Epidemiology, and End Results (SEER) database to explore the clinicopathologic features and risk factors of survival. To our knowledge, this is the largest population study to date to analyze UTUC with bone metastasis, which will provide evidence for clinical practice.

## Materials and methods

### Patient selection

Clinical data from the SEER database on UTUC with bone metastasis were obtained by using the case-listing session on the SEER*Stat version 8.3.9 software. We selected the primary tumor sites of UTUC by using the International Classification of Diseases for Oncology, 3rd edition (ICD-O-3) codes “C65.9-Renal pelvis” and “C66.9-Ureter.” Meanwhile, we set the SEER Combined Mets at DX-bone (2010+) to YES and finally identified the UTUC patients with bone metastasis. Patients with non-pathological diagnosis or death certificate diagnosis were excluded. In the current study, all UTUC patients had bone metastasis at initial diagnosis and were in M1 stage. After diagnosis, they received their treatments.

Information collected and also analyzed in from the SEER database includes race, gender, age at diagnosis, primary tumor site, pathological type, tumor size, treatment methods, visceral metastasis, marital status, vital status, survival time, and cause of death. Surgery or radiotherapy in the present study refers to treatment for primary tumor sites. According to previous studies (10, 11), OS and cancer-specific survival (CSS) were defined as the time from diagnosis till death due to any cause and due to the primary tumor, respectively.

### Statistical analysis

All statistical and descriptive analyses were performed by using the SPSS 23.0 software. Univariate Cox regression analyses were performed by analyzing race, gender, age at diagnosis, primary tumor site, pathological type, tumor size, treatment methods, visceral metastasis, marital status. Important variables from univariate analysis were incorporated for multivariate Cox regression analysis. Meanwhile, hazard ratio (HR) and their 95% confidence interval (95% CIs) were presented in univariate and multivariate analysis. Forest plots were drawn by GraphPad 8.0.1 and used to visually display the results of multivariate analysis. Kaplan-Meier method was applied to intuitively show the prognosis difference of each independent risk factor. Two-sided *p* value less than 0.05 was considered of significance.

## Results

### Demographic and clinical characteristics

**Figure 1:**
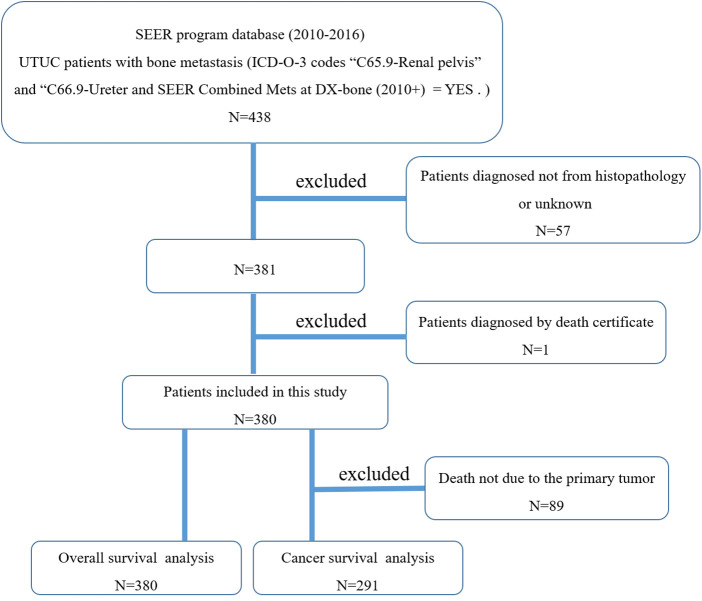
The flow chart for selection of study population. (SEER, Surveillance, Epidemiology, and End Results; ICD-O-3, international classification of diseases for oncology, 3rd edition; UTUC, upper-tract urothelial carcinoma).

Figure 1 showed the flow chart for selection of study population. In total, 380 cases who met the eligibility criteria were included in this study (Table 1), of which 230 males (60.5%) and 150 females (39.5%). More than four out of five (85.5%) of patients were white race. The mean and median age at diagnosis were 71 and 72 years, respectively. In terms of primary tumor site, 71.6% tumors were located in the renal pelvis, and 28.4% in the ureter. The majority of the patients (87.4%) were diagnosed as transitional cell papillomas and carcinomas. Other tumor types including (1) epithelial neoplasms, NOS, (2) squamous cell neoplasms, (3) adenomas and adenocarcinomas, (4) cystic, mucinous and serous neoplasms, (5) complex epithelial neoplasms, (6) complex mixed and stromal neoplasms, accounted for 12.6%. Tumor size <5 accounted for 24.5% and tumor size ≥5 accounted for 33.2%. Overall, only 29.7% of the patients received surgery, 37.4% received radiotherapy, and over half of patients (52.1%) had chemotherapy. There were 8(2.1%) patients with brain metastasis, 118 (31.1%) patients with liver metastasis, and 127 (133.4%) patients with lung metastasis. The 1-year OS and CSS rates of this research population were 23.8% and 26.6%, respectively. The median follow-up time for survivors was 2 years.

**Table 1:** Baseline characteristics of 380 UTUC with bone metastasis.

Variable	Value
**Race**	
White	325 (85.5%)
Black	19 (5.0%)
Others	36 (9.5%)
**Gender**	
Female	150 (39.5%)
Male	230 (60.5%)
**Age (years)**	
≤60	74 (19.5%)
>60	306 (80.5%)
Mean	71
Median	72
**Primary site**	
Renal pelvis	272 (71.6%)
Ureter	108 (28.4%)
**Pathological type**	
Transitional cell papillomas and carcinomas	332 (87.4%)
Others	48 (12.6%)
**Tumor size (cm)**	
<5	93 (24.5%)
≥5	126 (33.2%)
Unknown	161 (42.4%)
**Surgery**	
Yes	113 (29.7%)
No	267 (70.3%)
**Radiotherapy**	
Yes	142 (37.4%)
No	238 (62.6%)
**Chemotherapy**	
Yes	198 (52.1%)
No	182 (47.9%)
**Brain metastasis**	
No	359 (94.5%)
Yes	8 (2.1%)
Unknown	13 (3.4%)
**Liver metastasis**	
No	250 (65.8%)
Yes	118 (31.1%)
Unknown	12 (3.2%)
**Lung metastasis**	
No	241 (63.4%)
Yes	127 (33.4%)
Unknown	12 (3.2%)
**Marital status**	
Married	210 (55.3%)
Others	148 (38.9%)
Unknown	22 (5.8%)
**Dead**	
Yes	330 (86.8%)
No	50 (13.2%)
1-year OS rate	23.80%
1-year CSS rate	26.6%

UTUC, upper-tract urothelial carcinoma; OS, overall survival; CSS, cancer-specific survival.

## Univariate cox regression analysis

**Figure 2:**
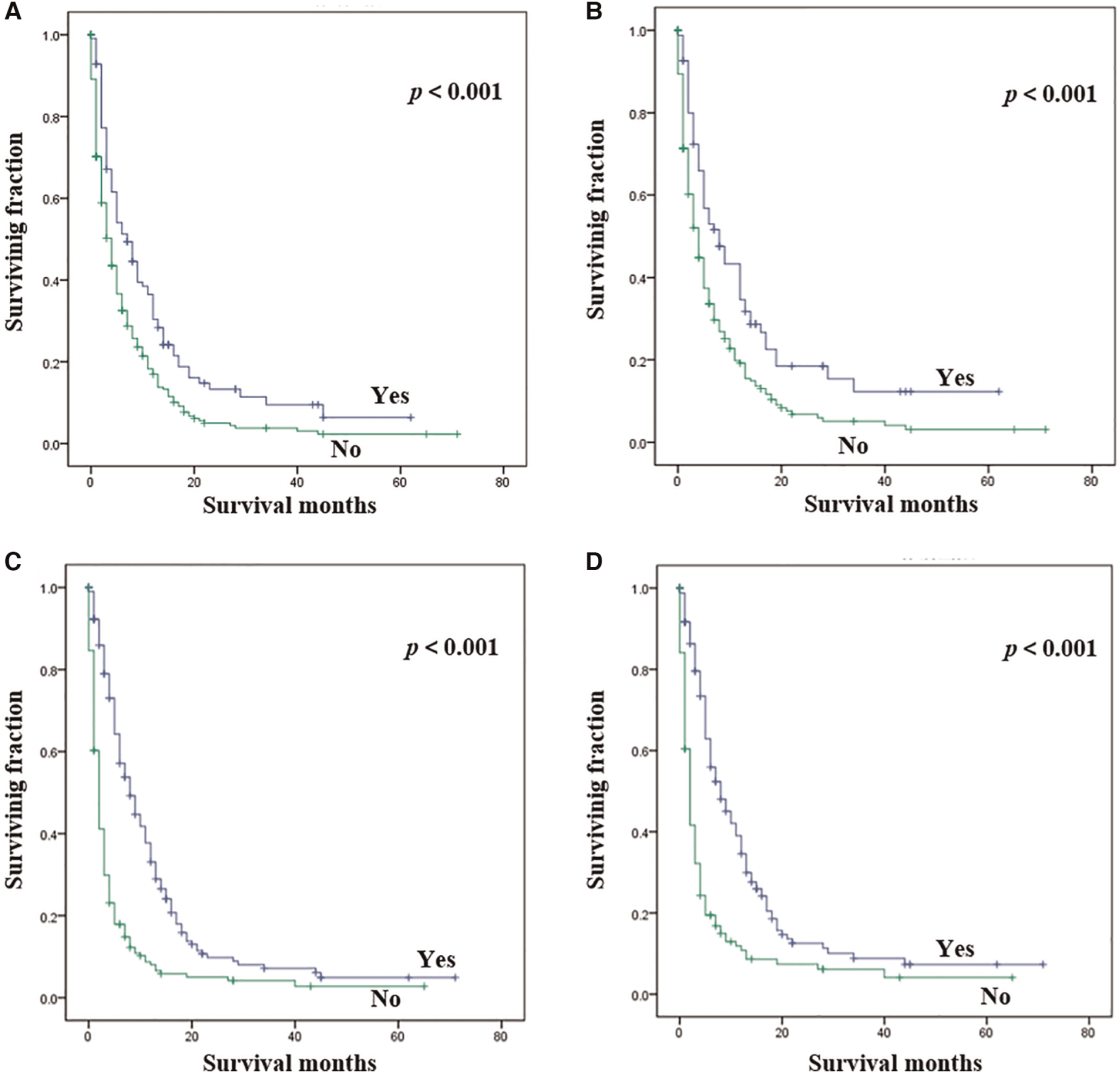
Kaplan-Meier method estimated OS and CSS in UTUC patients with bone metastasis stratified by treatment methods. (**A**) OS stratified by surgery; (**B**) CSS stratified by surgery; (**C**) OS stratified by chemotherapy; (**D**) CSS stratified by chemotherapy. (UTUC, upper-tract urothelial carcinoma; OS, overall survival; CSS: cancer-specific survival).

**Figure 3:**
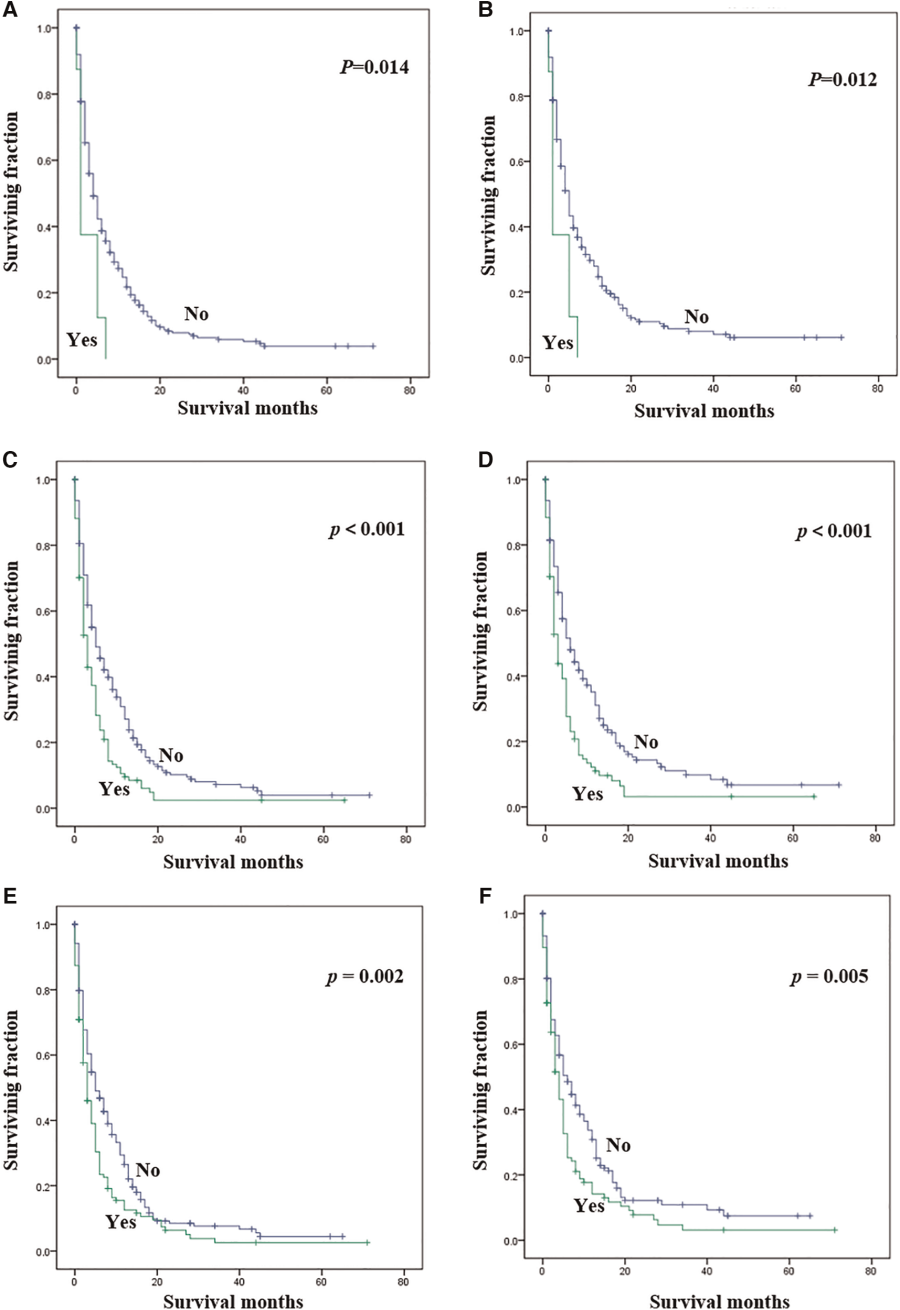
Kaplan-Meier method estimated OS and CSS in UTUC patients with bone metastasis stratified by visceral metastasis. (**A**) OS stratified by brain metastasis; (**B**) CSS stratified by brain metastasis; (**C**) OS stratified by liver metastasis; (**D**) CSS stratified by liver metastasis; (**E**) OS stratified by lung metastasis; (**F**) CSS stratified by lung metastasis. (UTUC, upper-tract urothelial carcinoma; OS, overall survival; CSS, cancer-specific survival).

**Figure 4:**
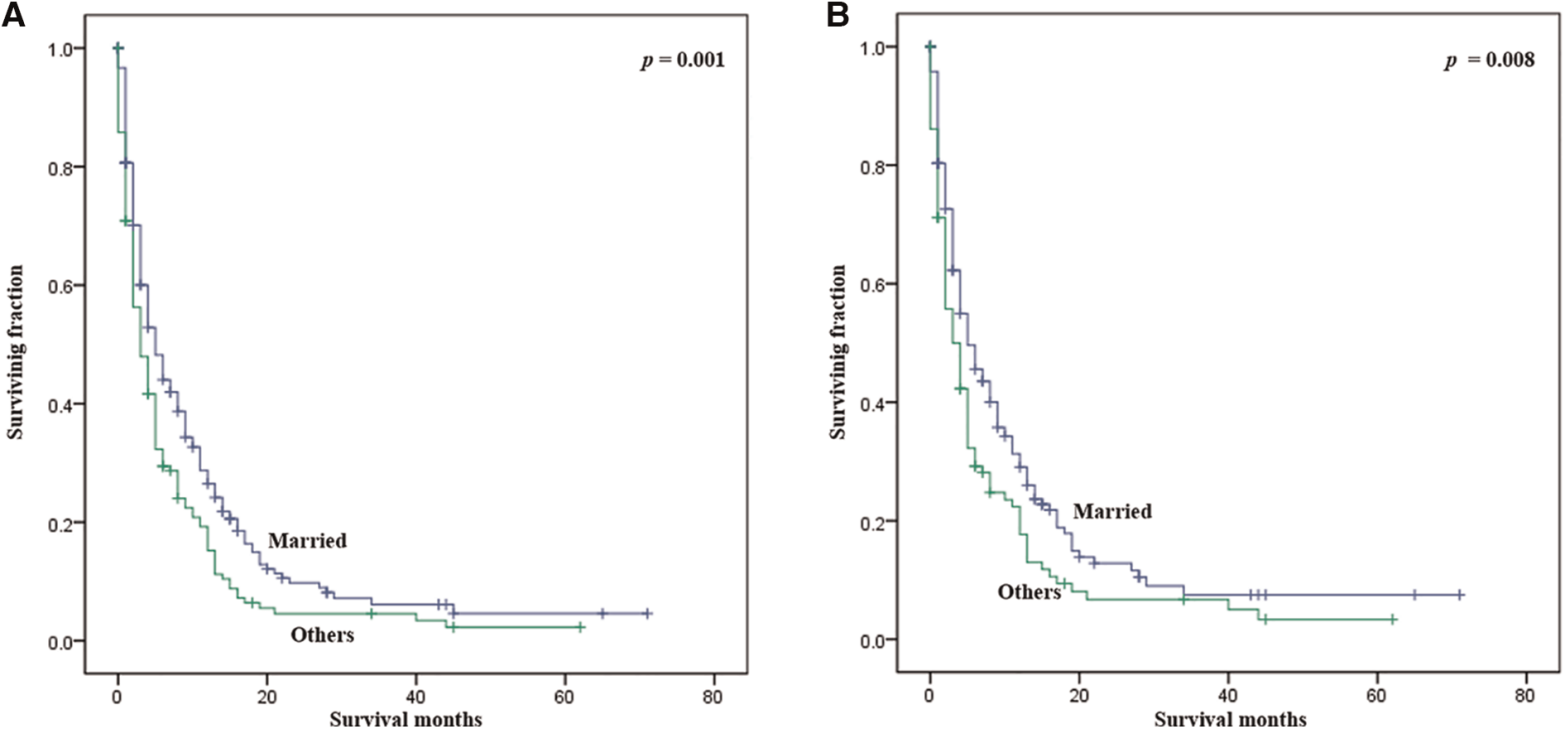
Kaplan-Meier method estimated OS and CSS in UTUC patients with bone metastasis stratified by marital status. (**A**) OS stratified by marital status; (**B**) CSS stratified by marital status. (UTUC, upper-tract urothelial carcinoma; OS, overall survival; CSS: cancer-specific survival).

Statistical results of univariate analysis of UTUC with bone metastasis were presented in Table 2. No significance on OS and CSS were observed in terms of race, gender, age, primary site, pathological type, tumor size, and radiotherapy. Patients receiving surgery and chemotherapy experienced the better OS and CSS. Kaplan-Meier plots of surgery and chemotherapy were shown in Figure 2. Patients with brain or liver or lung metastasis were significantly correlated with worse OS and CSS. Figure 3 showed the Kaplan-Meier plots of brain, liver and lung metastasis. Moreover, married patients had a significant prolonged prognosis (Figure 4).

**Table 2:** Univariate Cox analysis of variables in UTUC with bone metastasis.

Variable	OS		CSS	
	HR (95% CI)	*p*	HR (95% CI)	*p*
**Race**				
White	1		1	
Black	1.276 (0.770–2.115)	0.345	1.175 (0.655–2.107)	0.589
Others	0.880 (0.606–1.278)	0.502	0.799 (0.519–1.232)	0.31
**Gender**				
Female	1		1	
Male	0.845 (0.677–1.054)	0.135	0.864 (0.669–1.117)	0.265
**Age (years)**				
≤60	1		1	
>60	1.293 (0.978–1.709)	0.072	1.107 (0.817–1.501)	0.511
**Primary site**				
Renal pelvis	1		1	
Ureter	0.821 (0.645–1.045)	0.108	0.775 (0.584–1.028)	0.077
**Pathological type**				
Transitional cell papillomas and carcinomas	1		1	
Others	0.777 (0.557–1.084)	0.137	0.755 (0.517–1.104)	0.147
**Tumor size (cm)**				
<5	1		1	
≥5	1.065 (0.810–1.398)	0.653	0.965 (0.695–1.340)	0.833
**Surgery**				
Yes	1		1	
No	1.600 (1.255–2.040)	<0.001	1.691 (1.263–2.264)	<0.001
**Radiotherapy**				
Yes	1		1	
No	1.156 (0.924–1.445)	0.205	1.127 (0.870–1.460)	0.366
**Chemotherapy**				
Yes	1		1	
No	2.474 (1.978–3.095)	<0.001	2.315 (1.786–3.000)	<0.001
**Brain metastasis**				
No	1		1	
Yes	2.259 (1.115–4.579)	0.024	2.339 (1.148–4.763)	0.019
**Liver metastasis**				
No	1		1	
Yes	1.635 (1.292–2.069)	<0.001	1.719 (1.310–2.255)	<0.001
**Lung metastasis**				
No	1		1	
Yes	1.419 (1.127–1.788)	0.003	1.428 (1.096–1.861)	0.008
**Marital status**				
Married	1		1	
Others	1.417 (1.131–1.776)	0.002	1.410 (1.081–1.839)	0.011

UTUC, upper-tract urothelial carcinoma; OS, overall survival; CSS, cancer-specific survival.

## Multivariate cox regression analysis

**Figure 5:**
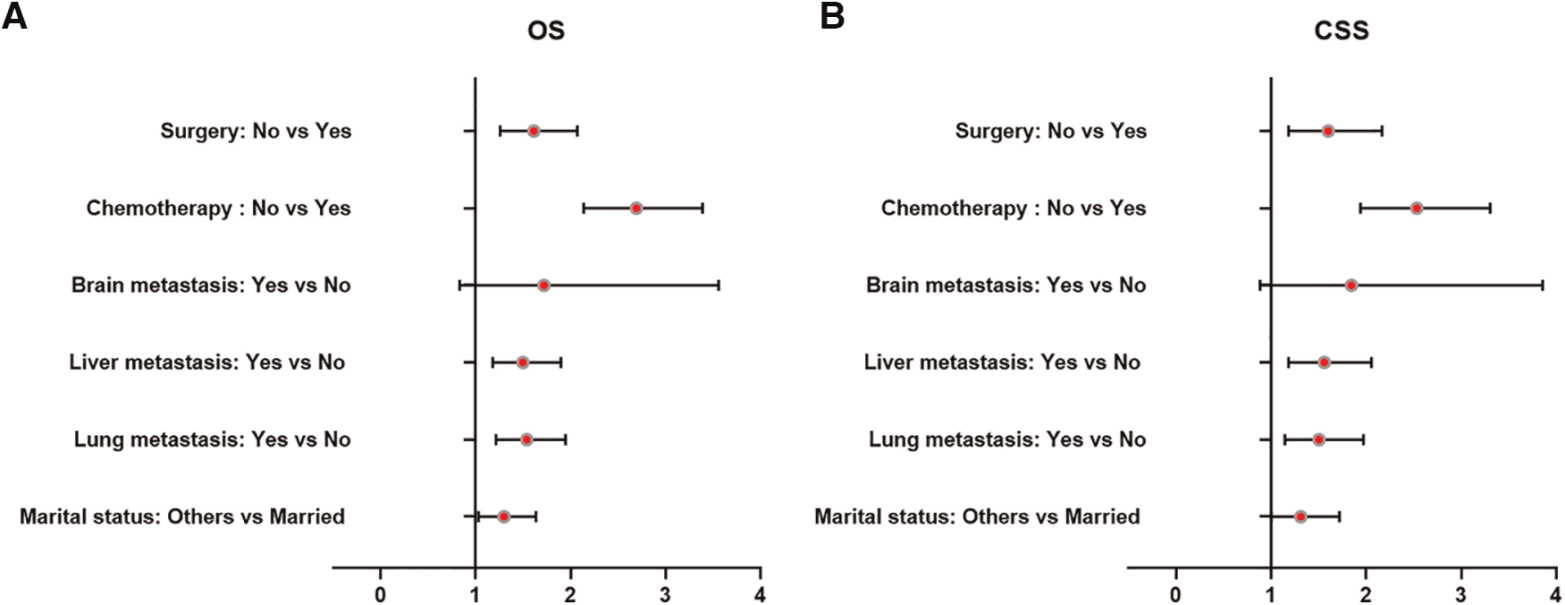
Forest plots of the predictors of OS (**A**) and CSS (**B**) in UTUC patients with bone metastasis. (UTUC, upper-tract urothelial carcinoma; OS, overall survival; CSS, cancer-specific survival).

Table 3 and Figure 5 presented statistical results of multivariate analysis of UTUC with bone metastasis. Age and primary tumor site were identified as independent risk factors of OS and CSS. Surgery and chemotherapy were the beneficial factors for OS and CSS. Liver and lung metastasis were significantly correlated with worse rates of OS and CSS. Brain metastasis and marital status did not confer a disadvantage to the survival for this population.

**Table 3:** Multivariate Cox analysis of variables in UTUC with bone metastasis.

Variable	OS		CSS	
	HR (95% CI)	*p*	HR (95% CI)	*p*
**Surgery**				
Yes	1		1	
No	1.613 (1.256–2.072)	<0.001	1.602 (1.184–2.167)	0.002
**Chemotherapy**				
Yes	1		1	
No	2.693 (2.139–3.391)	<0.001	2.533 (1.940–3.307)	<0.001
**Brain metastasis**				
No	1		1	
Yes	1.720 (0.831–3.561)	0.144	1.846 (0.884–3.858)	0.103
**Liver metastasis**				
No	1		1	
Yes	1.496 (1.179–1.898)	0.001	1.561 (1.185–2.057)	0.002
**Lung metastasis**				
No	1		1	
Yes	1.538 (1.214–1.948)	<0.001	1.503 (1.146–1.972)	0.003
**Marital status**				
Married	1		1	
Others	1.298 (1.030–1.635)	0.027	1.313 (1.002–1.720)	0.049

UTUC, upper-tract urothelial carcinoma; OS, overall survival; CSS, cancer-specific survival.

## Discussion

UTUC with bone metastasis is a relatively rare urothelial carcinoma, and little attention has been given to it (12). However, its incidence has been increasing in the past 30 years (13). Thus, it is necessary to investigate the independent survival predictors affecting UTUC with bone metastasis. To our knowledge, this is the largest population-based study to describe the demographic and clinical characteristics, and analyze the prognosis for UTUC patients with bone metastasis. The 1-year OS and CSS rates of 380 UTUC patients with bone metastasis were 23.8% and 26.6%, respectively, indicating a poor prognosis. Furthermore, our findings showed that surgery, chemotherapy, liver metastasis, lung metastasis, and marital status were significant independent predictors of survival, which provides a good assistance for clinicians and patients in treatment decisions.

Meaningful difference was not observed in terms of race, which was consistent with previous researches on UTUC (14, 15). However, some researchers reported that race was an independent prognostic factor of UTUC (16, 17). Although the male to female ratio in this study cohort was close to the overall UTUC patients (18), it was not a risk factor affecting the prognosis, which was in line with results of Mori et al. (19). However, Huang et al. (20) found that gender was a significant prognostic factor for all UTUC patients and females had significantly improved survival. Li et al. (17) identified that female patients had worse survival. The mean age of the study population was similar to that of UTUC patients overall. Previous studies identified older age as a poor survival predictor for UTUC (14, 17, 21). However, our study found that age was not correlated with survival among UTUC with bone metastasis. Primary tumor site was not a survival predictor of UTUC with bone metastasis, which was consistent with overall UTUC (22, 23). Additionally, Alessandro Veccia et al. (24) showed that tumor location in UTUC seems to be associated with outcomes, especially in the case of advanced disease. Although some studies suggest that tumor size was related to the prognosis of UTUC (17, 21), our present study revealed no association between tumor size and survival among UTUC patients with bone metastasis. Additionally, pathological type was not correlated with survival.

Univariate and multivariate analysis showed that marital status significantly impacted survival in UTUC with bone metastasis. Many studies have shown that marital status is an important factor affecting the prognosis of cancer patients, and married patients generally have a better prognosis due to the economic and emotional support (25–27). Married patients were more likely to gain curative treatment, high-quality care, and support of their spouse (28, 29). Thus, it is imperative to provide more support to those divorced, single, separated, and widowed patients. Overall, UTUC patients with bone metastasis exhibited different demographic and clinical characteristics compared with overall UTUC patients.

Simultaneous lung metastasis (33.4%) and liver metastasis (31.1%) were more common in UTUC patients with bone metastasis. Synchronous metastases significantly decreased the survival of patients with bone metastasis (10). Similarly, our study revealed that liver and lung metastases were independent prognostic factors of both OS and CSS. Brain metastasis is generally considered to be a poor prognostic factor in patients with bone metastasis (30). However, our multivariate analysis did not identify brain metastasis as an independent prognostic factor. It is possible that the number of patients with brain metastasis in this cohort was relatively small (8, 2.1%).

A recent meta-analysis revealed that perioperative chemotherapy for UTUC was beneficial for prolonging survival (31). Additionally, there is growing evidence that neoadjuvant chemotherapy has beneficial effects on pathologic downstaging of patients with UTUC (32). Surgical excision and chemotherapy were also suitable for UTUC patients with bone metastasis, which was consistent with previous mainstream treatments for patients with primary tumors (17, 33, 34). In advanced UTUC, radical nephroureterectomy (RNU) still remains the standard of care (35). Alberto Martini et al. (36) reported that neoadjuvant chemotherapy may be an option in patients with UTUC and bone metastasis. Although radiotherapy had no influence on survival of UTUC patients with bone metastasis, it may offer local control and reduce pain (37). Huang et al. (38) found that radiotherapy provided no significant benefit in survival of UTUC patients. Li et al. (17) reported that radiotherapy actually reduced the UTUC patient's prognosis. Thus, surgery and chemotherapy are recognized as optimal treatments to improve the survival of UTUC with bone metastasis.

The SEER database is a very powerful cancer research tool, which provides advantages for the study of patients with rare tumors. Of course, this study has some shortcomings. First, this study was an observational study design. Second, type of surgical treatment, radiotherapy and chemotherapy procedure, and immunotherapy were not defined in this cancer database. Third, the SEER database does not include information of local recurrence or distant metastasis during follow-up, which may affect the survival. Additionally, the SEER database does not contain information on the performance status of the patients and given this, it is usually not well characterized the decision to perform a treatment rather than others. Future randomized trials should be performed to solve the above problems.

## Conclusion

UTUC patients with bone metastasis had a poor prognosis, with 1-year OS and CSS rates 23.8% and 26.6%, respectively. Surgery and chemotherapy were beneficial for prolonging the survival of UTUC with bone metastasis. Liver and lung metastases were associated with worse prognosis. Additionally, patients with married status experienced better survival. Future randomized trials are needed to confirm these prognostic factors to better guide the management of such patients.

## Data Availability

The raw data supporting the conclusions of this article will be made available by the authors, without undue reservation.
